# Clinicopathologic findings in rats fed on the powder of *Caloncoba echinata *leaves

**DOI:** 10.30466/vrf.2018.89652.2170

**Published:** 2019-12-15

**Authors:** Christian Onwuchokwe Okorie-Kanu, Daniel Inua Edet, Patrick Emeka Aba, Onyinye Josephine Okorie-Kanu

**Affiliations:** 1 *Department of Veterinary Pathology, College of Veterinary Medicine, Michael Okpara University of Agriculture, Umudike, Nigeria; *; 2 *Department of Veterinary Physiology and Pharmacology, Faculty of Veterinary Medicine, University of Nigeria, Nsukka, Nigeria;*; 3 *Department of Veterinary Public Health and Preventive Medicine, Faculty of Veterinary Medicine, University of Nigeria, Nsukka, Nigeria.*

**Keywords:** Blood biochemistry, Caloncoba echinate, Hematology, Histopathology, Rats

## Abstract

This study investigated the toxic effects of dried pulverized *Caloncoba echinata* leaves on the hematology, blood biochemistry and vital organs of male albino rats. Twenty adult male rats were randomly divided into four groups of five rats each. Groups A, B and C were fed 25.00%, 15.00% and 5.00% of pulverized *C. echinata* leaves in feed respectively while group D was given normal feed for a four weeks period. Blood samples were collected at two weeks intervals for hematological and blood biochemistry analyses. Results showed a significant reduction of packed cell volume, hemoglobin concentration and total red blood cell counts in group B from week two to the end of the study. There was also a reduction of body weight and leukocytosis in groups A and B from week two to the end of the study. There was a significant reduction of albumin in group B when compared to the other groups after two weeks and a significant reduction in blood glucose concentration in group A after two weeks of the feeding of the leaves to the end of the study. Discrete areas of degeneration and necrosis of hepatocytes were observed in the rats of groups A and B and testicular atrophy in group B rats. It was concluded that feeding the rats with the pulverized *C. echinata* leaves led to a significant reduction of body weights and erythrocytic parameters, leukocytosis, hepatic and testicular injuries in the albino rats.

## Introduction

Toxins are present in almost every plant even in species that are commonly used for food in man and animals.^1^ Poisoning occurs when susceptible animals eat too much or quickly due to starvation or certain risk factors including drought which limits alternative forages, the introduction of stock into new areas with different plants, grazing on recently burned pastures and accidental intake with the other plants.^2^^,^^3^ Also, seasonal changes in toxin concentrations and the expansion of toxic populations as well as changes in plant palatability are important causes of plant poisoning. The degree of effect is influenced mainly by the availability and the abundance of toxic plants, climatic factors, and management of both livestock and pastures.^3^

Plant poisoning is one of the most important causes of economic loss in the livestock industry.^3^^,^^4^ Direct losses include deaths, weight loss, abortions, lengthened calving intervals, decreased efficiency and the other effects on the animals while indirect losses are expenses on fencing, herding, supplemental feeding, medical costs, manage-ment alterations, and loss of forage caused by efforts to prevent or minimize poisoning of livestock by plants.^4^ The global cost of plant-associated toxins may be many times larger as a result of additional human and animal poisonings when plant toxins contaminate prepared feeds, food, and herbal products.^1^

Many plants have been identified as poisonous to farm animals in Europe, Brazil, and South Africa with reports of their toxic effects.^1^^,^^5^^-^^7^ In Nigeria, except for a reported case of toxicity from death-camus (*Zygadenus chlorantus*) in Kayi-Yari village which resulted in death of 2256 sheep,^8^ reports of poisonous plants are mainly based on the information obtained from the villagers through questionnaires.^9^^-^^11^ *Caloncoba echinata* is a shrub indigenous to western Africa. It is claimed to possess some medicinal properties, however, it has not been well investigated.^12^ The only work on its anti-plasmodium properties was terminated because of its toxicity to the cells. *C. echinata* grows very luxuriantly especially during the dry season when most plants have withered making it readily available to herbivores. 

The suspected cases of plant poisoning of cows from the University herd, post mortem lesions, the abundance of *C. echinata* where the cows grazed and the presence of the plant’s leaves in rumens of some dead cows made us to further investigate the toxicity of the plant. Our preliminary investigation on the toxicity showed that the plant was relatively poisonous for mice.^13^ This study was, therefore, designed to investigate the toxicity of *C. echinata* leaves in rats.

## Materials and Methods

**Preparation of plant material.** *Caloncoba echinata* leaves were collected from Micheal Okpara University of Agriculture, Umudike, Abia State, Nigeria. The plant was identified by a taxonomist in the College of Natural Resources and Environmental management of Micheal Okpara University with herbarium No. MOUAU/VPP/ 2017/05. They were well dried under room temperature and ground.

**Experimental animals. **Twenty healthy nine-week-old male Sprague Dawley albino rats weighing between 95.00 - 103.00 g obtained from the research animal house of Department of Veterinary Pathology and Microbiology, University of Nigeria, Nsukka, Nigeria were used in the present study. The animals were acclimatized for two weeks in the clean cages at the research animal house. They were given feed (Grower’s mash containing maize, fish meal, palm kernel cake, salt, and some vitamins, Top Feed Nig. Ltd., Sapele, Nigeria) and water *ad libitum*. The principles of laboratory animal care (NIH publication No. 85-23, revised 1985) and specific national laws were observed and all experiments were approved by the University Committee on research ethics (MOUAU/EC/18/017).

The rats were randomly divided into four groups of five rats in each and the baseline values were determined after acclimatization. The feeds were pelleted and groups A, B, and C had their feeds incorporated with 25.00%, 15.00% and 5.00% of the pulverized leaves respectively, while group D was given a normal feed. The rats were observed for clinical signs in a 4-weeks period. 

**Hematology and blood biochemistry analyses. **Blood samples (4.00 mL) were collected via the retrobulbar plexus, and one mL of each sample was transferred into a sterile tube with ethylene diamine tetracetic acid (EDTA) for hematology and three mL was transferred into another sterile tube without anti-coagulant. The blood samples were kept at room temperature for 30 min and centrifuged at 3000 *g* for 10 min and then serums were harvested and stored in the refrigerator for blood biochemistry examinations on weeks 0, 2 and 4.

Hematological examinations were carried out immediately after blood collection while the blood biochemical examinations were carried out within 12 hr after sampling. Packed cell volume (PCV) and hemoglobin concentration (Hb) were determined using the micro hematocrit and cyanomethemoglobin methods respectively.^14^^,^^15^ Red blood cell (RBC) and total white blood cell (WBC) counts were determined by the hemo-cytometer method. Erythrocytic indices were obtained using the standard formula while the differential WBC count was determined by the Leishman method.^14^^,^^15^ The plasma protein was determined using a refractometer (VEEGEE CLX-1; Nova Tech, Culver City, USA).

**Clinical biochemistry methods.** All analytes (Table 1) were determined using serum samples and Quimica Clinica Aplicada test kits (Quimica Clinica Aplicada, Tarragona, Spain) and a Cole-Palmer 1200 spectro-photometer (Cole-Palmer Instrument Co., Vernon Hills, USA) while the blood glucose level was determined using Accu-chek Active^®^ Glucometer (Roche Diagnostics GmbH, Mannheim, Germany). 

**Table 1:** Clinical biochemistry methods

**Analytes**	**Method**
**Alanine amino transferase **Reitman-Frankel^16^	
**Aspartate amino transferase **Reitman-Frankel^16^	
**Alkaline phosphatase **	Phenolphthalein monophosphate ^17^^,^^18^
**Total protein**	Direct Biuret^19^
**Albumin**	Bromocresol green^20^^,^^21^
**Globulin**	Total protein-albumin^22^
**Total cholesterol**	Enzymatic colorimetric^23^
**Blood urea nitrogen **	Modified Berthlot-Searcy^24^
**Creatinine** **Glucose**	Modified Jaffe^25^ Glucose oxidase^26^

**Histopathology**. At the end of the study, the animals were sacrificed and vital organs including liver, kidney, spleen, pancreas, lungs, and testes were removed. Tissue samples were fixed in buffered normal saline. The fixed tissues were dehydrated by passing them through graded alcohol for 2 hr in each alcohol percentage. They were then removed and put in chloroform overnight for clearing. The following morning, the tissues were infiltrated with paraffin wax. They were then embedded with paraffin wax using an embedding machine. Tissue sections of 5.00 μm of thickness were fixed on slides and stained with Hematoxylin and Eosin (H & E).^27^

**Statistical Analysis. **Data generated were subjected to analysis of variance (ANOVA) and variant means were separated using the least significant difference (LSD) method using SPSS statistical package (version 16.0; SPSS Inc., Chicago, USA). A *p* value less than 0.05 was considered significant.

## Results

**Clinical Signs. **No clinical signs were observed in rats. 

**Hematology. **Hematological results are presented in Table 2. There was no significant reduction in all the hematological parameters determined on day 0. There was significant reduction of the PCV, Hb and RBC values (*p *< 0.05) in group B rats when compared with the other groups on weeks 2 and 4. There were significantly increased WBC counts (*p *< 0.05) in groups A and B values when compared with groups C and D on week 2, but on week 4 only group B was significant. There was increased lymphocyte count in groups A and B when compared with groups C and D but only significant difference was observed in group A) on week 2 (*p *< 0.05).

Blood biochemistry results are presented in Table 3. There was no significant reduction in all the blood biochemistry parameters determined on day 0. However, there were significant reductions in serum total proteins and albumin (*p *< 0.05) and a significant increase in globulin values (*p *< 0.05) in group B rats when compared with the other groups on week 2. There was also a significant reduction in blood glucose concentration (*p *< 0.05) in group A of rats when compared with the control on weeks 2 and 3.

**Fig. 1:**
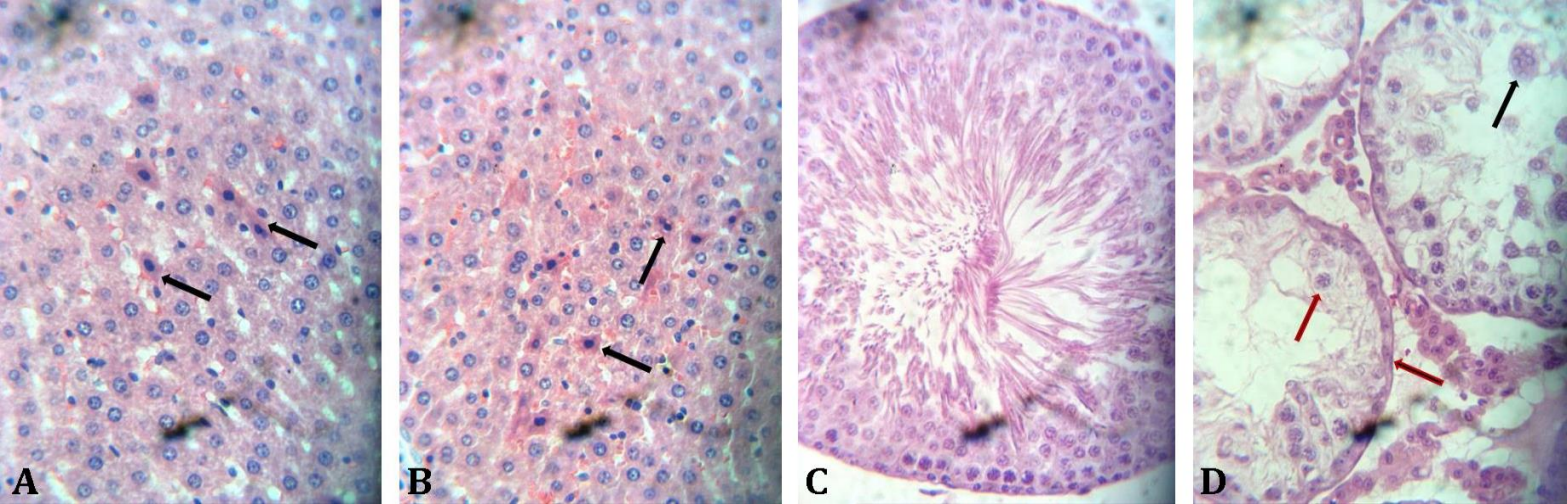
**A) **Discrete degeneration and necrosis of hepatocytes (arrows); **B)** Discrete degeneration and necrosis of hepatocytes (arrows); **C)** Normal seminiferous tubules; and **D)** Testicular atrophy and degeneration are shown by multinucleated spermatids (black arrow) and degeneration and necrosis (red arrows) of spermatogonia and spermatocytes (H & E, 400×)

**Histopathology. **There were degeneration and necrosis of hepatocytes in groups A and B rats and no lesions in the rats of groups C and D. There were testicular atrophy, degeneration, and necrosis of spermatids and spermatocytes in group B and no lesions seen in the animals of other groups (Fig. 1).

**Table 2:** Hematological profile (mean ± standard error) of Sprague Dawley albino rats after the administration of pulverized leaves of *Caloncoba echinata*

**Parameters**	**Week 2**				**Week 4**			
	**Group A**	**Group B**	**Group C**	**Group D**	**Group A**	**Group B**	**Group C**	**Group D**
**PCV (%)**	43.80 ± 0.60^a^	40.40 ± 0.37^b^	43.80 ± 1.36^c^	43.10 ± 0.91^c^	41.60 ± 0.29^a^	38.70 ± 0.20^b^	44.20 ± 0.64^c^	45.10 ± 0.53^c^
**HbC (g dL** ^-1^ **)**	14.91 ± 0.17^a^	13.53 ± 0.17^b^	14.78 ± 0.71^a^	14.63 ± 0.27^ab^	14.28 ± 0.24^a^	13.25 ± 0.31^b^	14.33 ± 0.17^a^	14.29 ± 0.16^a^
**RBC (10** ^6^ ** µL** ^-1^ **)**	7.81 ± 0.17^a^	7.38 ± 0.08^b^	7.97 ± 0.16^a^	7.60 ± 0.07^ab^	7.91 ± 0.18^a^	7.11 ± 0.10^b^	8.03 ± 0.20^a^	7.80 ± 0.17^a^
**MCV (fL)**	54.39 ± 2.17	54.39 ± 1.00	54.96 ± 1.51	56.73 ± 1.04	52.73 ± 1.29	54.41 ± 0.71	55.21 ± 1.61	54.90 ± 0.64
**MCH (pg)**	19.33 ± 0.57	18.37 ± 0.34	18.57 ± 0.85	19.25 ± 0.29	18.06 ± 0.54	18.62 ± 0.48	17.90 ± 0.54	18.37 ± 0.51
**MCHC (g dL** ^-1^ **)**	34.06 ± 0.51	33.24 ± 0.49	33.72 ± 1.00	33.63 ± 0.60	34.24±0.45	34.23±0.74	32.44±0.50	31.72±0.63
**WBC (10** ^3^ ** µL** ^-1^ **)**	18.18 ± 1.55^a^	15.23 ± 1.51^a^	11.53 ± 0.32^b^	11.53 ± 0.76^b^	14.85 ± 1.55^a^	13.12 ± 0.86^b^	13.18 ± 1.22^b^	12.05 ± 0.98^b^
**Neutrophil**	3.89 ± 0.28	4.22 ± 1.06	2.70 ± 0.15	2.40 ± 0.43	2.77 ± 0.18	2.61 ± 0.37	2.56 ± 0.24	2.41 ± 0.24
**Lymphocyte**	13.87 ± 1.53^a^	10.77 ± 0.82^b^	8.59 ± 0.38^b^	9.29 ± 0.70^b^	11.55 ± 1.40	10.35 ± 0.81	10.63 ± 1.12	9.09 ± 1.10
**Monocyte**	0.15 ± 0.04	0.17 ± 0.02	0.19 ± 0.07	0.06 ± 0.03	0.14 ± 0.04	0.13 ± 0.05	0.09 ± 0.06	0.05 ± 0.03
**Eosinophil**	0.03 ± 0.03	0.07 ± 0.05	0.00 ± 0.00	0.05 ± 0.05	0.06 ± 0.04	0.03 ± 0.03	0.06 ± 0.06	0.02 ± 0.02
**Basophil**	0.03 ± 0.03	0.04 ± 0.04	0.02 ± 0.02	0.00 ± 0.00	0.00 ± 0.00	0.00 ± 0.00	0.00 ± 0.00	0.00 ± 0.00
**Plasma protein (g dL** ^-1^ **)**	8.00 ± 0.17	8.16 ± 0.25	8.16 ± 0.07	8.48 ± 0.21	7.68 ± 0.10	7.38 ± 0.25	7.90 ± 0.32	7.72 ± 0.19

PCV= Packed cell volume; HbC= Hemoglobin concentration; RBC= Red blood cell; MCV= Mean corpuscular volume; MCH= Mean corpuscular hemoglobin; MCHC= Mean corpuscular hemoglobin concentration, WBC= White blood cell.

^abc ^Different superscripts in each row indicate significant differences between the groups (*p *< 0.05).

**Table 3:** Blood biochemistry profile (mean ± standard error) of Sprague Dawley albino rats after the administration of pulverized leaves of *Caloncoba echinata*

**Parameters**	**Week 2**				**Week 4**			
	**Group A**	**Group B**	**Group C**	**Group D**	**Group A**	**Group B**	**Group C**	**Group D**
**ALT (IU L** ^-1^ **)**	38.44 ± 0.26	38.55 ± 0.27	38.78 ± 0.16	38.17 ± 0.14	39.46 ± 0.22	39.61 ± 0.20	39.23 ± 0.21	38.91 ± 0.24
**AST (IU L** ^-1^ **)**	45.06 ± 0.31	45.36 ± 0.16	45.34 ± 0.12	45.15 ± 0.04	44.96 ± 0.11	44.74 ± 0.05	44.67 ± 0.20	44.83 ± 0.14
**ALP (IU L** ^-1^ **)**	210.44 ± 6.13	203.64 ± 2.98	205.58 ± 3.10	201.53 ± 0.87	226.71 ± 3.80	210.43 ± 13.02	213.00 ± 6.86	208.39 ± 8.62
**TP (g dL** ^-1^ **)**	5.71 ± 0.24	6.19 ± 0.36	6.02 ± 0.10	6.19 ± 0.14	6.42 ± 0.28	6.15 ± 0.39	5.89 ± 0.25	5.94 ± 0.16
**Albumin (g dL** ^-1^ **)**	3.17 ± 0.28^ab^	2.75 ± 0.37^a^	3.58 ± 0.15^b^	3.27 ± 0.28^ab^	3.46 ± 0.24	3.21 ± 0.27	3.37 ± 0.22	3.63 ± 0.17
**Globulin (g dL** ^-1^ **)**	2.54 ± 0.29^ab^	3.47 ± 0.26a	2.44 ± 0.17^b^	2.92 ± 0.21^ab^	2.76 ± 0.30	2.94 ± 0.31	2.51 ± 0.36	2.11 ± 0.17
**Glucose (mg dL** ^-1^ **)**	67.20 ± 3.73^a^	75.40 ± 3.19^ab^	75.80 ± 2.36^ab^	83.40 ± 4.46^b^	66.80 ± 4.55^a^	79.00 ± 1.76^bc^	71.80 ± 0.97^ab^	82.40 ± 3.83^c^
**Total Chol (mg dL** ^-1^ **)**	86.09 ± 13.88	100.87 ± 7.58	106.09 ± 10.43	95.65 ± 9.12	92.17 ± 7.06	92.17 ± 4.43	86.56 ± 6.15	96.65 ± 6.15
**Total Bil (mg dL** ^-1^ **)**	2.32 ± 0.11	2.16 ± 0.14	2.38 ± 0.14	2.07 ± 0.16	2.37 ± 1.35	2.33 ± 0.17	2.20 ± 0.17	2.25 ± 0.09
**BUN (mg dL** ^-1^ **)**	31.74 ± 1.66	32.26 ± 1.68	28.65 ± 2.81	30.19 ± 2.75	28.64 ± 1.55	27.23 ± 1.71	27.10 ± 1.61	31.61 ± 1.61
**Creatinine (mg dL** ^-1^ **)**	0.67 ± 0.00	0.67 ± 0.00	0.67 ± 0.00	0.67 ± 0.00	0.67 ± 0.00	0.67 ± 0.00	0.60 ± 0.07	0.60 ± 0.07

ALT= Alanine aminotransferase; AST= Aspartate aminotransferase; ALP= Alkaline phosphatase; TP= Total protein; Chol= cholesterol, Bil= Bilirubin; BUN= Blood urea nitrogen.

^abc ^Different superscripts in a row indicate significant difference between the groups in each collection (*p *< 0.05).

## Discussion

The results of the packed cell volume (PCV), red blood cell (RBC) counts and hemoglobin (Hb) concentration showed a concentration and time-related significant reduction in the mean values of these parameters in group B without any significant change in the mean corpuscular values. These findings differed from the observations from an earlier report in mice although the study lasted for only one week.^13^ These results imply that the administration of *C. echinata *led to a normocytic, normochromic reduction in the erythrocyte parameters of the rats. These findings are common in all species when the hematopoietic system is adversely affected.^28^^,^^29^ The reductions in the erythrocyte parameters occur normally due to the excessive blood loss, the excessive red blood cell destruction or decreased erythrocyte production as a result of the exposure to the toxic substances or irradiation.^29^^,^^30^

The normocytic, normochromic reduction in erythro-cyte parameters recorded in this study suggests impairment or suppression of erythrocyte production in the bone marrow due to some metabolites of the plant. The leukocytosis may be attributed to the lesions caused by plant metabolites in several organs especially liver and also following an inflammatory reaction. This is as a result of the mobilization of marginating neutrophils from the peripheral blood and the bone marrow storage pool.^15^^,^^30^^,^^31^ Neutrophils are primarily phagocytic cells and therefore are associated with infectious diseases or tissue injury.^15^ The reduced albumin concentration could have resulted from liver injury which led to liver insufficiency coupled with the reduced intake of feed. Hypoproteinemia follows reduced synthesis of protein as a result of chronic hepatopathies; malabsorption caused by enteritis, tumors, and parasitism; increased loss of protein consequent upon glomerular disorder and starvation or malnutrition.^29^^,^^31^ Albumin is the most abundant protein found in plasma and therefore their reduction always causes hypo-proteinemia. The reduced blood glucose concentration might have resulted from liver injury as seen in groups A and B and reduced feed intake probably caused by the unpalatability of the feed. The injuries observed in the hepatocytes may also be caused by the metabolites of the plant. Hepatic injury was also observed in our earlier study.^13^ Testicular atrophy and degeneration showed by smaller diameter seminiferous tubules, necrosis and degeneration of spermatocytes and spermatids may be effects of the plant metabolites. The causes of testicular atrophy and degeneration include advance in age, chemotherapy, heat, metal compound toxicosis, nutritional disorders, plants toxins, and stress.^32^ The presence of secondary metabolites in the plants had been associated with the pharmacologic, toxicologic and other biologic effects of medicinal plants. These findings are in agreement with the cytotoxic effect of cycloartane isolated from the fruit of *C. glauca*,^7^ a closely related plant to *C. echinata*.

The pathologic effects of the plant on the haematology, blood biochemistry, liver and testis were more in group B compared to group A that received a higher percentage of the plant. This may be due to the unpalatability of the feed with the high inclusion of the plant resulting in the reduced intake and consequent higher intake of water which could have diluted the little quantity consumed by the rats. The changes in the hematologic parameters, blood biochemistry, and presence of degenerations and necrosis in hepatic cells and seminiferous tubules may be associated with the administration of pulverized leaves of *C. echinata*. 
